# Knockdown of autophagy-related protein 5, ATG5, decreases oxidative stress and has an opposing effect on camptothecin-induced cytotoxicity in osteosarcoma cells

**DOI:** 10.1186/1471-2407-13-500

**Published:** 2013-10-26

**Authors:** Mario G Hollomon, Nancy Gordon, Janice M Santiago-O’Farrill, Eugenie S Kleinerman

**Affiliations:** 1 Division of Pediatrics, The University of Texas MD Anderson Cancer Center, Houston, TX 77054, USA; 2 Department of Biology, Texas Southern University, Houston, TX 77004, USA

**Keywords:** Autophagy, Osteosarcoma, Camptothecin, Oxidative stress

## Abstract

**Background:**

Autophagy induction can increase or decrease anticancer drug efficacy. Anticancer drug-induced autophagy induction is poorly characterized in osteosarcoma (OS). In this study, we investigated the impact of autophagy inhibition on camptothecin (CPT)-induced cytotoxicity in OS.

**Methods:**

Autophagy-inhibited DLM8 and K7M3 metastatic murine OS cell lines were generated by infection with lentiviral shRNA directed against the essential autophagy protein ATG5. Knockdown of ATG5 protein expression and inhibition of autophagy was confirmed by immunoblot of ATG5 and LC3II proteins, respectively. Metabolic activity was determined by MTT assay and cell viability was determined by trypan blue exclusion. Acridine orange staining and immunoblotting for LC3II protein expression were used to determine autophagy induction. Oxidative stress was assessed by staining cells with HE and DCFH-DA followed by flow cytometry analysis. Mitochondrial membrane potential was determined by staining cells with TMRE followed by flow cytometry analysis. Immunoblotting was used to detect caspase activation, Parp cleavage and p53 phosphorylation.

**Results:**

Autophagy inhibition caused a greater deficit in metabolic activity and cell growth in K7M3 cells compared to DLM8 cells. K7M3 cells exhibited higher basal autophagy levels than DLM8 cells and non-transformed murine MCT3 osteoblasts. Autophagy inhibition did not affect CPT-induced DNA damage. Autophagy inhibition decreased CPT-induced cell death in DLM8 cells while increasing CPT-induced cell death in K7M3 cells. Autophagy inhibition reduced CPT-induced mitochondrial damage and CPT-induced caspase activation in DLM8 cells. Buthionine sulfoximine (BSO)-induced cell death was greater in autophagy-competent DLM8 cells and was reversed by antioxidant pretreatment. Camptothecin-induced and BSO-induced autophagy induction was also reversed by antioxidant pretreatment. Significantly, autophagy inhibition not only reduced CPT-induced oxidative stress but also reduced basal oxidative stress.

**Conclusions:**

The results of this study indicate that autophagy inhibition can have an opposing effect on CPT-induced cytotoxicity within OS. The cytoprotective mechanism of autophagy inhibition observed in DLM8 cells involves reduced CPT-induced oxidative stress and not reduced DNA damage. Our results also reveal the novel finding that knockdown of ATG5 protein reduces both basal oxidative stress and drug-induced oxidative stress.

## Background

Autophagy is a lysosomal-dependent process that occurs at low basal levels to support cellular homeostasis. During periods of nutrient deprivation, autophagy degrades intracellular proteins to serve as substrates for ATP generation. Autophagy also carries out housekeeping activities such as clearing the cell of damaged organelles and proteins that result from ordinary cellular metabolic activity. For example, damaged mitochondria are selectively targeted for autophagy, thus reducing the release of pro-apoptotic mediators into the cytosol and subsequent cell death [1]. Therefore, basal levels of autophagy are necessary for cellular homeostasis.

Autophagic activity above basal levels (hereafter referred to as autophagy induction) is induced by anticancer drug treatment. While autophagy inhibition both increases anticancer drug efficacy [2] and decreases anticancer drug efficacy [3,4], the majority of studies indicate that autophagy inhibition increases anticancer drug efficacy, suggesting that autophagy induction is a protective response to anticancer drug treatment. However, unrestrained drug-induced autophagy induction can lead to cell death [5].

Osteosarcoma (OS) is the most prevalent bone tumor in children. Despite recent advances in the understanding of the molecular basis of OS and new therapeutic approaches, the mortality rate has declined only modestly. Autophagy modulation as adjuvant therapy to established anticancer therapies is currently being investigated in clinical trials, but not in OS [6]. The use of autophagy modulation as adjuvant therapy in OS may prove beneficial. However, before considering such, the impact of anticancer drug-induced autophagy induction on cytotoxicity in OS must be better characterized.

In this study, we investigated the impact of autophagy inhibition on camptothecin (CPT)-induced cytotoxicity in OS cells. Camptothecin induces cell death by inhibiting topoisomerase I resulting in DNA single-strand breaks and subsequent cell death [7]. Here, we show that autophagy inhibition has an opposing impact on CPT-induced cytotoxicity in two metastatic murine OS cell lines. Autophagy inhibition in K7M3 cells increased sensitivity to CPT. In contrast, autophagy inhibition in DLM8 cells decreased sensitivity to CPT. The mechanism of autophagy inhibition-mediated protection in DLM8 cells appeared to be reduced CPT-induced oxidative stress and a reduction in both mitochondrial damage and caspase activation. To our knowledge, this is the first report of an opposing effect of anticancer drug treatment on cytotoxicity in autophagy-inhibited OS cells. Furthermore, we were unable to locate any other report of autophagy inhibition decreasing anticancer drug-induced oxidative stress.

## Methods

### Antibodies and reagents

Camptothecin was purchased from ChemWerth (Woodbridge, CN). LC3 and ATG5 antibodies were purchased from Novus Biologicals (Littleton, CO). Cleaved PARP, total p53, phospho p53, cleaved caspase-3 and cleaved caspase-9 antibodies were purchased from Cell Signaling Technology, Inc. (Danvers, MA). Pan-caspase inhibitor was purchased from Enzo Life Sciences (Farmingdale, NY). Ripa lysis buffer was purchased from Santa Cruz Biotechnology, Inc. (Santa Cruz, CA). Acridine orange, 3-(4,5-dimethylthiazolyl-2)-2,5-diphenylthetrazolium bromide (MTT) reagent, Bafilomycin A1 and actin antibody were purchased from Sigma Aldrich (St. Louis, MO). Buthionine sulfoximine (BSO) was purchased from Acros Organics (Morris Plains, NJ). N-acetyl cysteine (NAC) was purchased from Calbiochem (Billerica, MA). Fetal bovine serum (FBS) was purchased from Atlanta Biologicals (Lawrenceville, GA). DMEM cell culture medium and supplements, dihydroethidium (HE), 2′,7′-dichlorofluorescein diacetate (DCFH-DA), carbonyl cyanide chlorophenylhydrozone (CCCP) and tetramethylrhodamine, ethyl ester (TMRE) were purchased from Invitrogen (Carlsbad, CA).

### Cell lines and cell culture

DLM8 [8] and K7M3 [9] are metastatic murine OS cell lines. MC3T3 is a non-transformed murine osteoblast cell line [10]. Cells were cultured in Dulbecco’s modified eagle medium (DMEM) containing 10% FBS supplemented with antibiotic, non-essential amino acids, glutamine, sodium pyruvate and cultured in an incubator maintained at 5% CO_2_ and 37°C. Prior to experimentation, cells were karyotyped and tested for mycoplasm contamination. Cells were treated with CPT as indicated in figure legends. Treatments were based on sensitivity of each cell line to CPT. Where appropriate, cells were treated with BSO to induce oxidative stress and NAC to counter oxidative stress.

Lentiviral shRNA (Open Biosystems, Rockford, IL) targeted to autophagy-related protein-5 (ATG5) RNA was used to knockdown ATG5 protein expression. Two separate ATG5 knockdown cell lines were generated for each cell line using two different lentiviral shRNA sequences [TRCN0000099431, TRCN0000099433]. Briefly, lentivirus was produced by transfecting 293T cells with 7 ug/ml transfer plasmid [TRCN0000099431 or TRCN0000099433], 5 ug/ml psPAX2 (packaging plasmid) and 4 ug/ml pMD2. G (envelop plasmid). Forty-eight hours after 293T cell transfection, supernatant containing lentivirus was collected and immediately used for infection or stored at -80°C. For infection, 2 ml of supernatant containing lentivirus was added to each well of a 6-well plate containing 1×10^5^ cells. Cells were incubated with lentivirus for 12 h and next transferred to a 75 mm flask. Assessment of ATG5 protein knockdown was determined when cells were approximately 70% confluent. Both ATG5 knockdown cell lines showed similar responses to CPT treatment. Control cells were infected with lentivirus containing empty shRNA vector. Cells treated with empty shRNA vector are hereafter referred to as autophagy-competent. ATG5 protein knockdown cells are hereafter referred to as autophagy-inhibited.

### Cell growth, cell metabolic activity and cell viability determination

Cell growth was determined by seeding 4×10^4^ cells per well in a 12-well plate followed by cell count at 48 h. Metabolic activity was assessed by MTT assay. Metabolic activity converts yellow MTT reagent to a purple formazan. Color intensity is indicative of metabolic activity. MTT reagent (1mg/ml) was added to cells (3×10^3^ cells per well) and incubated for 1 h at 37°C followed by solubilization of formazan with DMSO followed by determination of formazan color intensity with a microplate reader set at absorbance reading 570 nm. Absorbance readings of autophagy-inhibited groups were compared to autophagy-competent groups which were normalized to one hundred percent. To determine cell viability, 4×10^4^ cells per well were seeded in 12-well plates. Following CPT treatment, cell viability was determined by trypan blue exclusion assay using an automated cell counter (Vi-Cell, Beckman Coulter, Miami, FL). Cells restricting trypan blue entry were considered viable.

### Acidic vesicular organelle (AVO) staining

Acridine orange freely diffuses the membranes of cells and organelles. Inside acidic vesicles, acridine orange is protonated and fluoresces bright red. Increased red fluorescence indicates increased acidic vesicular organelle (AVO) formation [11]. Following CPT treatment, cell culture medium was removed from the cells and replaced with cell culture medium containing 1ug/ml acridine orange and incubated for 20 min at 37°C. Cells were then removed, washed twice and fluorescence immediately analyzed using the FL3 channel of a FACSCalibur flow cytometer (Becton Dickinson, San Jose, CA).

### Western blot

Following drug treatment, supernatant and cells were collected and centrifuged at 300 g for 5 min at 4°C. The resultant pellet was lysed with RIPA lysis buffer containing protease and phosphatase inhibitor cocktail and centrifuged at 10,000 g for 10 min at 4°C. Supernatants were then collected and total protein was determined by BioRad reagent (BioRad Laboratories, Hercules, CA). Unless otherwise indicated, 30 ug of protein were resolved in SDS-polyacrylamide gels (SDS-PAGE) and transferred onto nitrocellulose membranes (BioRad Laboratories, Hercules, CA). Membranes were blocked with 5% nonfat milk then incubated with antibodies against ATG5, LC3, cleaved caspase-9, cleaved caspase-3, total p53, phospho p53 or cleaved PARP. Membranes were then washed and incubated with appropriate secondary antibody conjugated to HRP (GE Healthcare Life Sciences, Piscataway, NJ). Following secondary antibody incubation, membranes were washed and signal detected with ECL detection reagent (GE Healthcare Life Sciences, Piscataway, NJ). Beta-actin protein expression served as a protein loading control.

### Oxidative stress determination

Following drug treatment, cell culture medium was removed from the cells and replaced with cell culture medium containing 5 uM dihydroethidium (HE) or 5 uM 2′,7′-dichlorofluorescein diacetate (DCFH-DA) and incubated for 20 min at 37°C to assess superoxide anion (^.^O_2_^-^) and hydrogen peroxide (H_2_O_2_) levels, respectfully. Cells were then removed, washed twice and fluorescence immediately analyzed using a FACSCalibur flow cytometer (Becton Dickinson, San Jose, CA). HE freely diffuses the plasma membrane and is reduced by intracellular ^.^O_2_^-^ resulting in a red fluorescence. Intracellular DCFH-DA reacts with H_2_O_2_ to give a green fluorescence. Increased HE and DCFH-DA fluorescence indicates increased ^.^O_2_^-^ and H_2_O_2_ presence, respectively.

### Mitochondrial membrane potential (ΔΨm)

Tetramethylrhodamine ethyl ester perchlorate (TMRE) preferentially stains mitochondria producing red fluorescence and is used as an indicator of mitochondrial membrane potential (ΔΨm). Decreased TMRE fluorescence is indicative of ΔΨm depolarization and ΔΨm depolarization is associated with release of pro-apoptotic mediators [12]. Following CPT treatment, cells were incubated with 25 ng/ml TMRE for 20 min at 37°C to assess ΔΨm. The protonophore carbonylcyanide m-chlorophenylhydrozone (CCCP) was used as a positive control for ΔΨm depolarization and to test TMRE staining efficiency.

### Statistical analysis

Results are presented as means ± S.E.M. Experimental data were analyzed using 2-tailed Student *t* test. P values less than 0.05 were considered statistically significant.

## Results

### CPT decreases metabolic activity, cell growth and induces cell death

**Figure 1:**
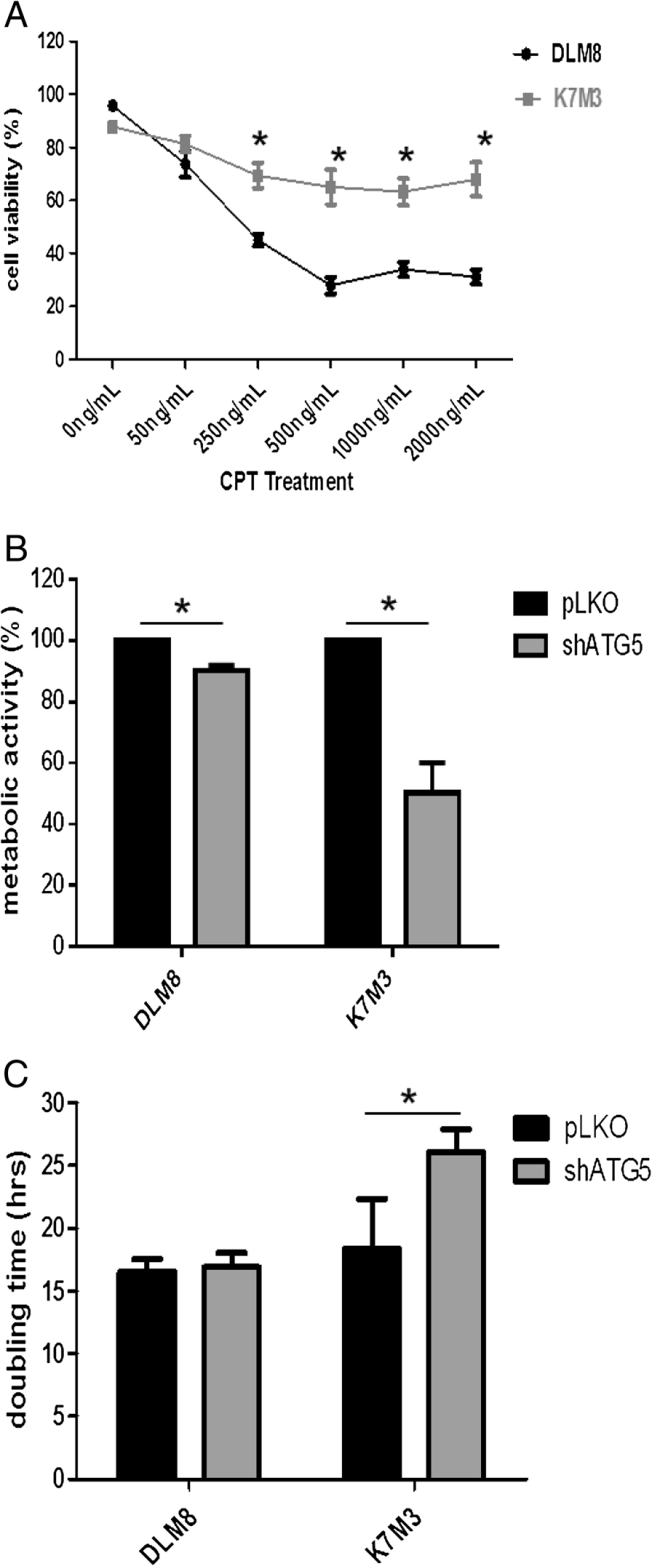
**Camptothecin decreases cell viability and metabolic activity. A**, Camptothecin-induced cell death. DLM8 and K7M3 cells were cultured in 12-well plates and treated with CPT as indicated for 48 h. Cell viability was determined by trypan blue exclusion assay. *, p < 0.05, compared with same treatment group. **B**, Impact of autophagy inhibition on metabolic activity. Cells were grown in a 96-well plate and allowed to grow in normal media to approximately 70% confluency. MTT assay was used to determine metabolic activity. Control values were set to one hundred percent. *, p < 0.05. **C**, Impact of autophagy inhibition on cell growth. Cells were grown in 12-well plates in normal media followed by cell count at 48 h. *, p < 0.05. Data represents the results of at least three independent experiments, ± SE. p < 0.05 was considered significant.

To begin this study, we assessed CPT-induced cytotoxicity in two metastatic murine OS cell lines. Camptothecin caused a dose-dependent decrease in cell viability in DLM8 and K7M3 cells (Figure 1A). Basal level of autophagy is associated with metabolic homeostasis; therefore, we determined if autophagy inhibition affected metabolic activity or cell growth. Autophagy inhibition significantly reduced both metabolic activity and cell growth in K7M3 cells (Figure 1B and C).

### CPT induces apoptosis and autophagy

**Figure 2:**
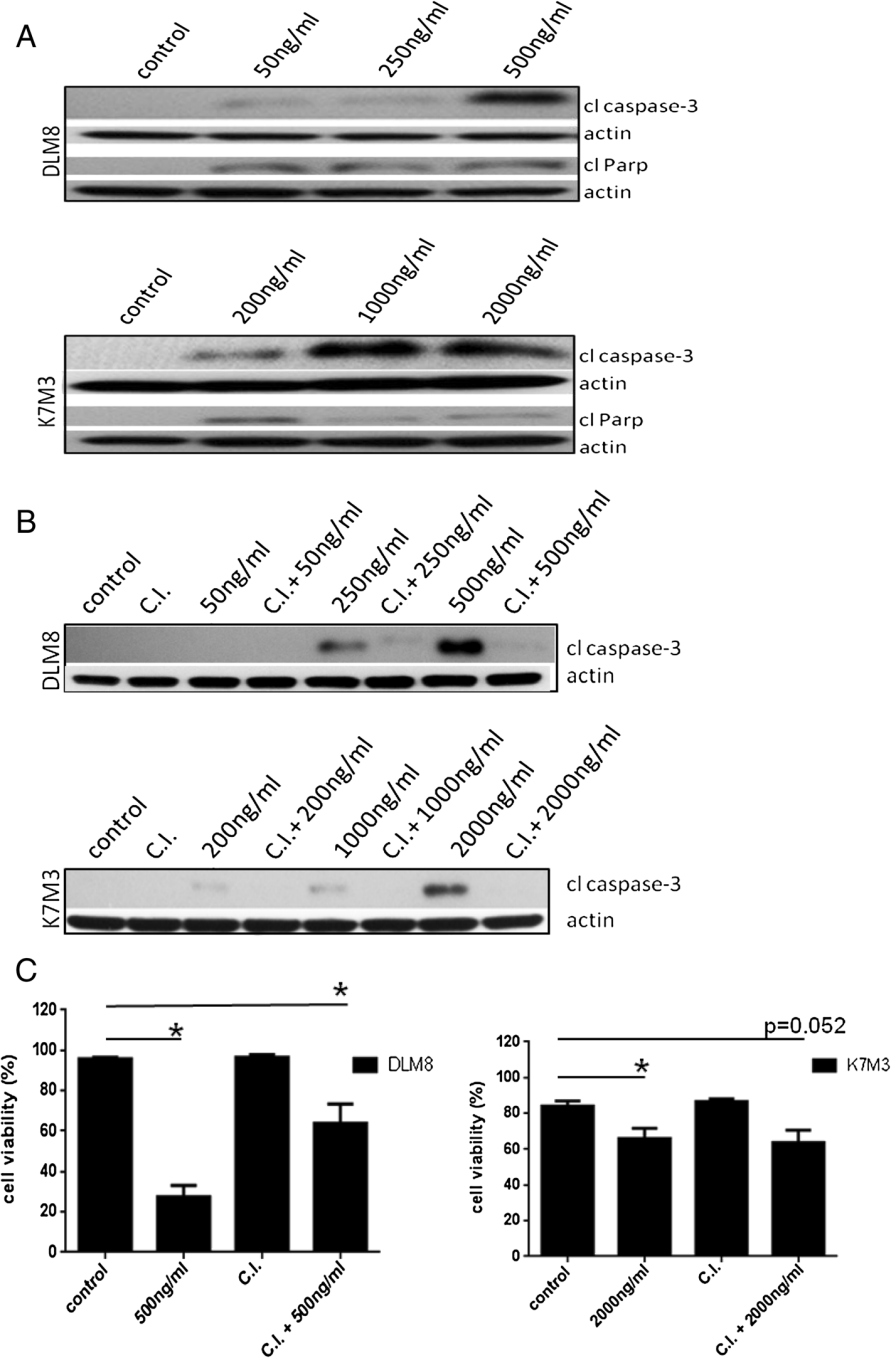
**Camptothecin induces caspase activation.** Cells were treated with no drug, or CPT or caspase inhibitor plus CPT at doses as indicated for 48 h. Following drug treatment, cells were lysed and cell lysate immunoblotted for cleaved caspase-3 and cleaved Parp protein expression. **A**, Cleaved caspase-3 and cleaved PARP protein expression in wildtype DLM8 and K7M3 cells. **B**, Pan caspase inhibitor blocks caspase-3 activation. Cells were treated with CPT doses indicated in figure for 48 h. Treatment doses were based on cell sensitivity to CPT. **C**, Caspase inhibition reverses CPT-induced cell death in DLM8. Wildtype DLM8 and K7M3 cells were pretreated with a pan-caspase inhibitor for 2 h followed by CPT treatment for 48 h. Control group received no drug and an additional group received CPT only. Cell viability was determined by trypan blue exclusion assay. *, p < 0.05, compared with control group. Data represents the results of at least three independent experiments, ± SE. p < 0.05 was considered significant. Actin served as a protein loading control. Immunoblots are representative of immunoblots from at least two independent experiments.

**Figure 3:**
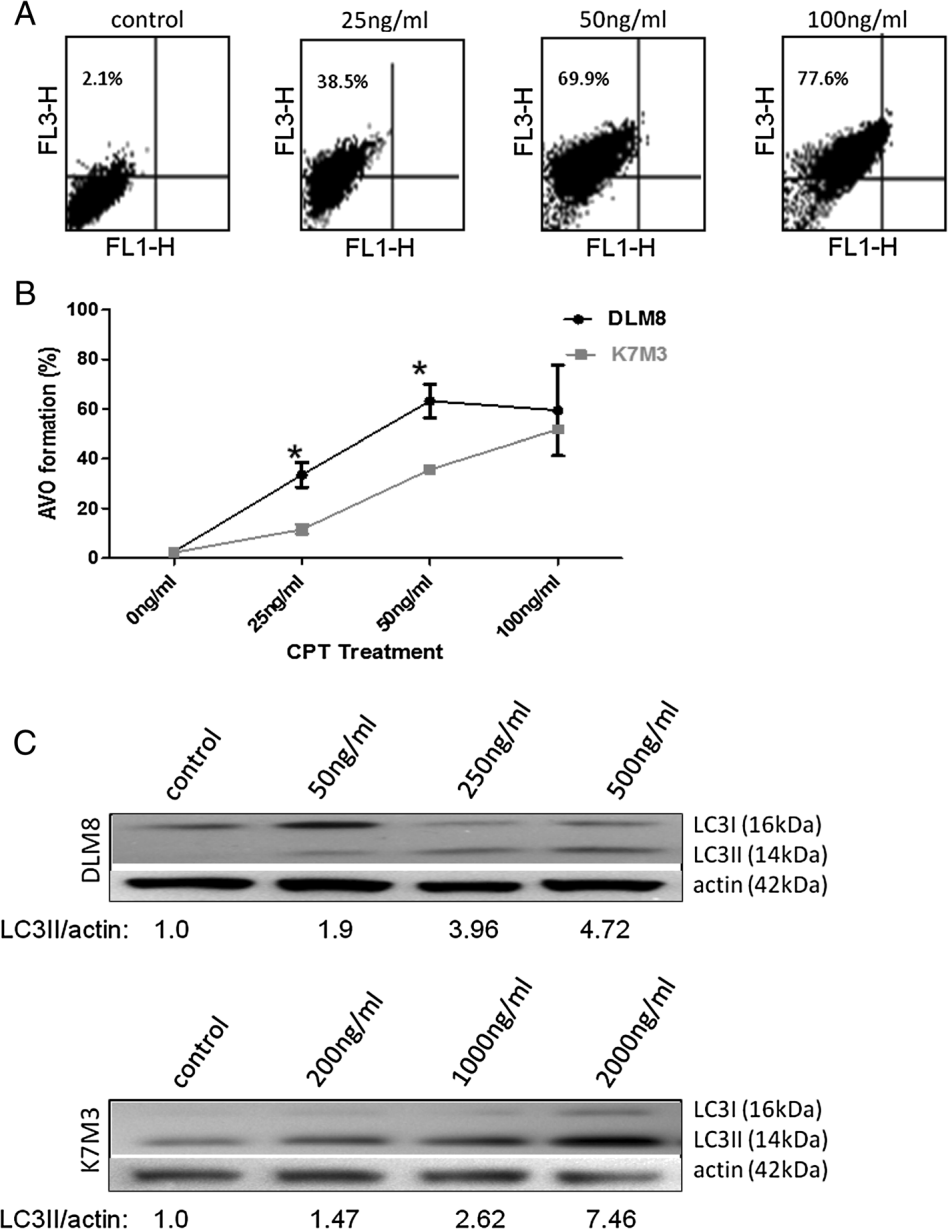
**Camptothecin increases autophagic activity.** Following 48 h CPT treatment, cells were incubated with the lysosomotropic agent acridine orange and fluorescence analyzed by flow cytometry. **A**, Representative flow cytometry analysis of acidic vesicular organelle (AVO) formation in wildtype DLM8 cells. **B**, Graph representation of CPT-induced AVO formation in wildtype DLM8 and K7M3 cells. *, p < 0.05, compared with same treatment group. **C**, LC3I/LC3II protein expression. Following 48 h CPT treatment, cells were lysed and cell lysate immunoblotted for LC3I/LC3II protein expression. Increased LC3II expression is indicative of autophagy induction. The expression of treatment group LC3II/actin ratio was determined by densitometry and compared to control group LC3II/actin ratio which was normalized to the arbitrary value of one. Treatment group LC3II expression was normalized to control actin levels as needed. 30ug of protein were loaded for DLM8 LC3I/LC3II determination while only 7.5ug of protein were loaded for K7M3 LC3I/LC3II determination. Actin served as a protein loading control. Data represents the results of three independent experiments, ± SE. p < 0.05 was considered significant. Immunoblot is representative of immunoblots from three independent experiments.

To determine CPT-induced apoptosis we assessed markers of apoptosis. Cleaved caspase-3 and cleaved PARP (Figure 2A) with accompanying cell death indicated CPT-induced apoptotic cell death. Pre-treatment with pan-caspase inhibitor blocked caspase-3 activation in both cell lines (Figure 2B) and reversed CPT-induced cell death in DLM8 cells but not in K7M3 cells (Figure 2C). Acidic vesicular organelle accumulation was determined to screen for increased autophagic activity following CPT treatment. Camptothecin treatment significantly increased AVO production in DLM8 and K7M3 cells (Figure 3A and B). Autophagy induction was confirmed by LC3II immunoblot. During autophagy induction, LC3I is converted to LC3II. LC3II protein expression increased in both cell lines following CPT treatment, confirming increased autophagic activity (Figure 3C). It is important to note that to measure LC3II protein levels, 30 ug of total protein from DLM8 were loaded to a SDS-PAGE gel, while only 7.5 ug of total protein from K7M3 were loaded. Thirty micrograms of total protein from K7M3 resulted in saturation of the membrane which prevented detection of differences in protein expression between treatment groups. Camptothecin-induced autophagy induction was also confirmed by assessment of a second autophagy marker p62 (Additional file 1: Figure S1). Reduced p62 protein expression is indicative of autophagy induction. Wildtype cells were treated with Bafilomycin A1 to determine the functional status of autophagy. Bafilomycin A1 inhibits autophagosome and lysosome fusion causing an increase in LC3II accumulation. Bafilomycin A1 caused an increase in LC3II accumulation compared to non-treated cells in both cell lines (Additional file 2: Figure S2), indicating that autophagy flux was functional in both cell lines.

### Knockdown of ATG5 protein expression has an opposing impact on cell viability in DLM8 and K7M3 OS cells

**Figure 4:**
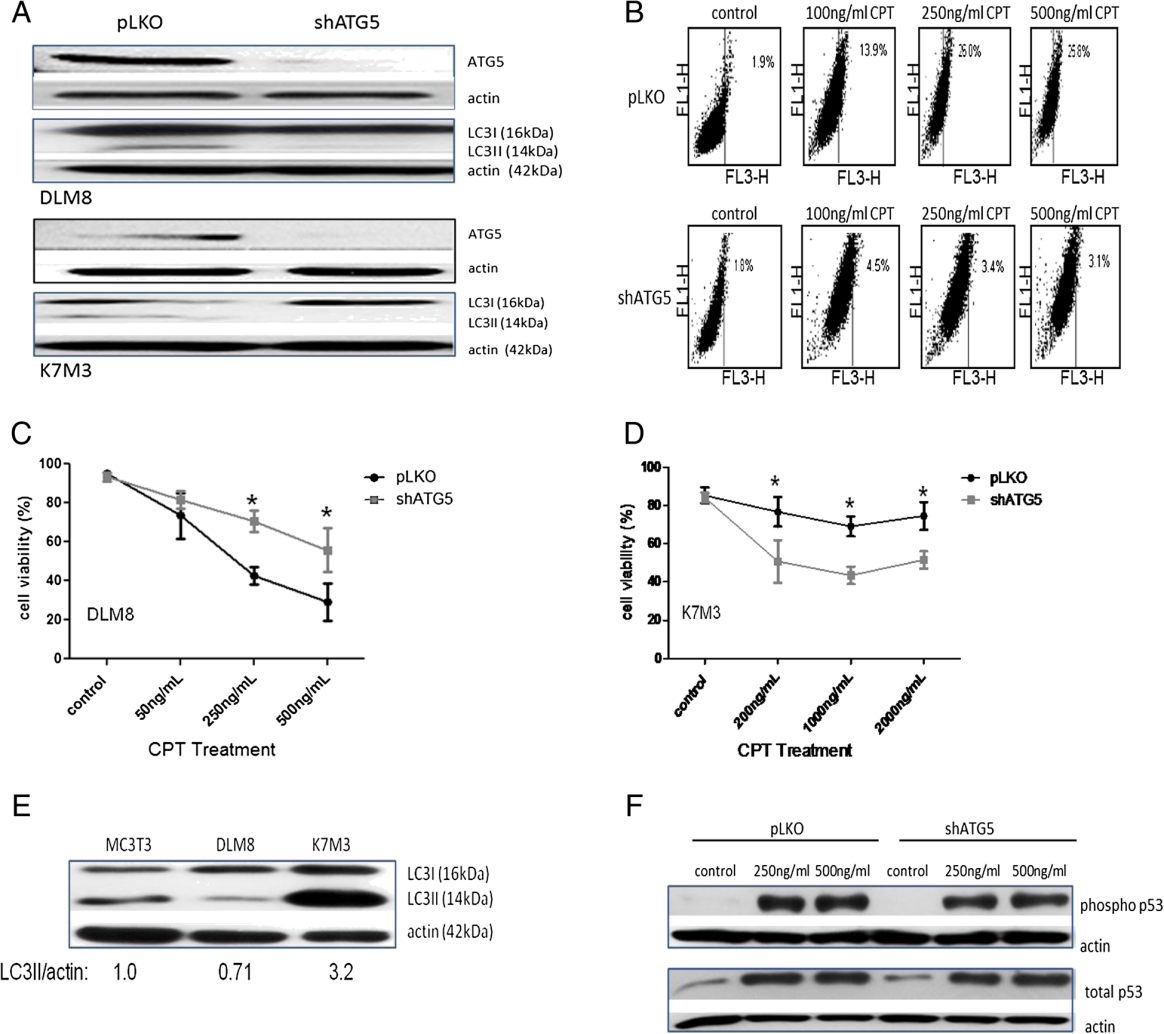
**Autophagy inhibition has an opposing impact on CPT-induced cell death. A**, ATG5 protein levels in DLM8 and K7M3 cells following shRNA-mediated knockdown of ATG5. Cells were infected with lentivirus containing empty shRNA vector or lentiviral shRNA targeted against ATG5 mRNA. Following infection, cells were lysed and total protein collected. To confirm ATG5 protein knockdown and autophagy inhibition, total protein was immunoblotted for ATG5 and LC3I/C3II protein levels, respectively. Actin served as a protein loading control. **B**, Acidic vesicular organelle (AVO) formation. Autophagy-competent and autophagy-inhibited DLM8 cells were treated with CPT for 24 h followed by assessment of AVO formation. Impact of autophagy inhibition on cell death in **C**, DLM8 and **D**, K7M3 OS cells. Autophagy-competent and autophagy-inhibited DLM8 and K7M3 cells were treated with CPT as indicated for 48 h. Following drug treatment, cell viability was assessed by trypan blue exclusion. *, p < 0.05, compared with same treatment group. **E**, Basal levels of autophagy in MC3T3, DLM8 and K7M3 cells. Cells were untreated and allowed to grow to approximately 70% confluency. Cells were then collected, lysed and total protein immunoblotted for LC3I/LC3II. 30ug of protein were loaded for each cell line. Actin served as a protein loading control. **F**, Phosphorylation of p53 in DLM8 cells. Cells were treated with CPT as indicated for 24 h. Following CPT treatment, cells were lysed and cell lysate probed for phospho p53 and total p53 protein expression. Data represents the results of at least three independent experiments, ± SE. p < 0.05 was considered significant. Immunoblots are representative of immunoblots from two independent experiments.

Autophagy was inhibited by knocking down the expression of essential autophagy protein ATG5. Knockdown of ATG5 protein expression and its impact on autophagy inhibition were confirmed by immunoblot of ATG5 and LC3II, respectively (Figure 4A). Knockdown of ATG5 reduced CPT-induced AVO formation, thus validating AVO as a reliable screen for autophagy induction (Figure 4B). Knockdown of ATG5 protein expression in DLM8 cells decreased CPT-induced cell death. In contrast, knockdown of ATG5 protein expression in K7M3 cells increased CPT-induced cell death (Figure 4C and D). Basal levels of autophagy were higher in K7M3 cells compared to DLM8 cells and a nontransformed osteoblast cell line, suggesting increased dependence of K7M3 on autophagy for metabolic homeostasis (Figure 4E). Camptothecin treatment induced similar level of phosphorylation of p53 at Ser15 in both autophagy-competent and autophagy-inhibited DLM8 cells, indicating similar levels of CPT-induced DNA damage (Figure 4F).

### Autophagy inhibition decreases CPT-induced oxidative stress and buthionine sulfoximine (BSO)-induced cell death

**Figure 5:**
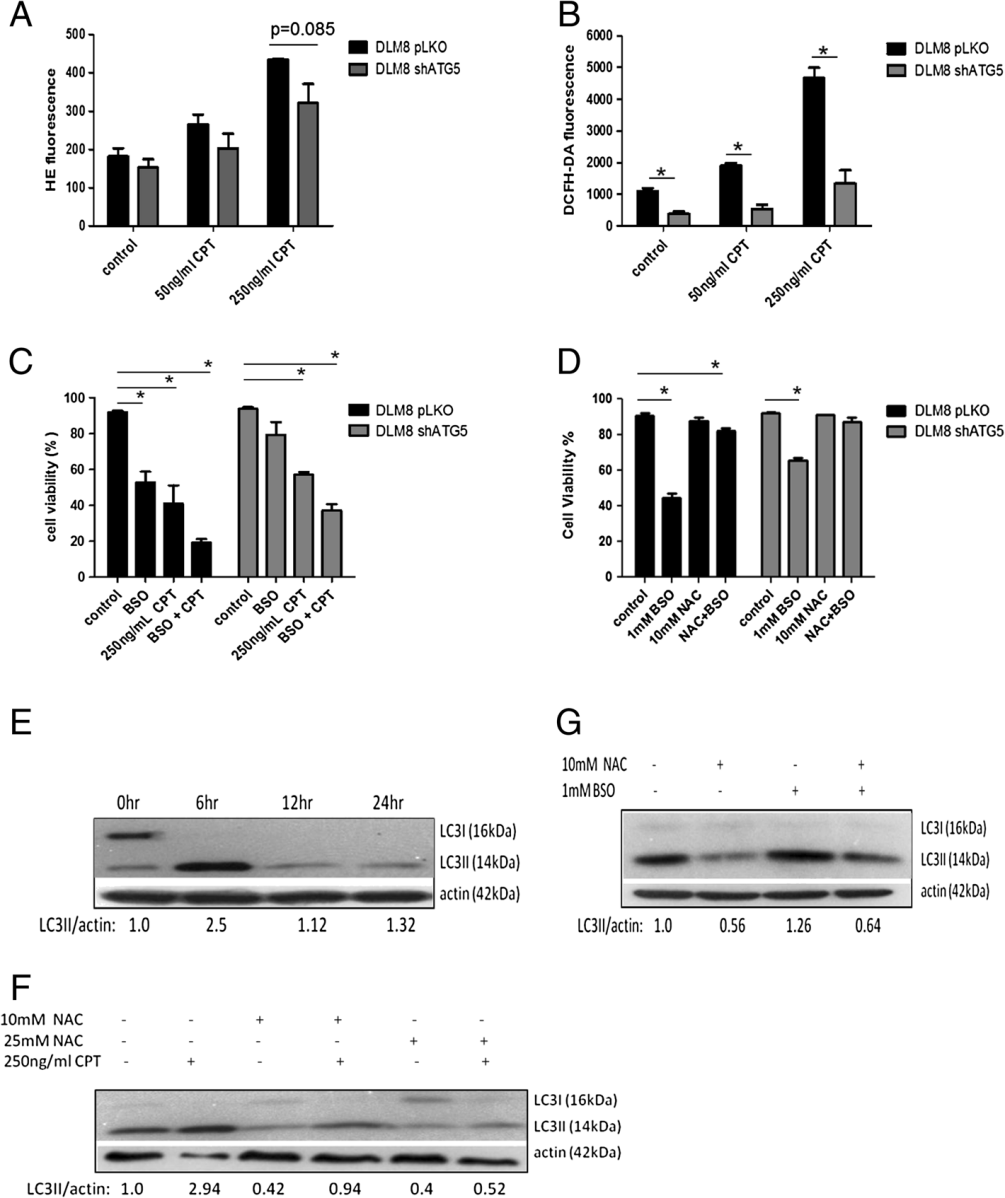
**Autophagy inhibition decreases CPT-induced oxidative stress and buthionine sulfoximine (BSO)-induced cell death.** Cells were treated with CPT for 24 h followed by incubation with HE or DCFH-DA. **A**, Camptothecin-induced ^.^O_2_^-^. *, p < 0.05. **B**, Camptothecin-induced H_2_O_2_. *, p < 0.05. **C**, Autophagy-competent cells are more sensitive to BSO-induced cell death. Cells were pretreated with 1mM BSO for 2 h followed by 48 h CPT treatment. Cells received a second 1mM BSO treatment 12 h into the CPT treatment. Following CPT treatment, cell viability was determined by trypan blue exclusion. *, p < 0.05, compared with control group. **D** The antioxidant NAC reverses BSO-induced cell death. Cells were pretreated with NAC for 2 h prior to BSO treatment. Cells received a second 1mM BSO treatment 12 h into the BSO treatment. Following 48 h BSO treatment, cell viability was determined by trypan blue exclusion. *, p < 0.05, compared with control group. **E**, BSO treatment increases LC3II levels in autophagy-competent DLM8 cells. Cells were treated with 1mM BSO for times indicated in figure. **F**, NAC pretreatment inhibits CPT-induced autophagy induction in autophagy-competent DLM8 cells. Cells received no drug, NAC, CPT or combination as indicated in figure for 48 h. For combination groups, cells were pretreated with NAC for 2 h. **G**, NAC pretreatment inhibits BSO-induced autophagy induction in autophagy-competent DLM8 cells. Cells received no drug, NAC, 1mM BSO or combination for 6 h. For combination group, cells were pretreated with NAC for 2 h. Following drug treatment, cells were lysed and total protein immunoblotted for LC3I/LC3II protein expression. Actin served as a protein loading control. Data represents the results of three independent experiments, ± SE. p < 0.05 was considered significant. Immunoblots are representative of immunoblots from at least two independent experiments.

To investigate the impact of autophagy inhibition on CPT-induced oxidative stress, HE and DCFH-DA probes were used to access ^.^O_2_^-^ and H_2_O_2_ levels, respectively. The level of CPT-induced ^.^O_2_^-^ and H_2_O_2_ generation was greater in autophagy-competent DLM8 cells (Figure 5A and B). To determine if autophagy-competent DLM8 cells were more sensitive to oxidative stress in general, cell viability was assessed in autophagy-competent and autophagy-inhibited DLM8 cells following BSO or combination treatment of BSO and CPT. Buthionine sulfoximine inhibits synthesis of the endogenous antioxidant glutathione [13], thus increasing oxidative stress levels. Autophagy-competent DLM8 cells were more sensitive to BSO-induced cell death and the cotreatment of BSO and CPT caused greater cell death in autophagy-competent DLM8 cells compared to autophagy-inhibited DLM8 cells (Figure 5C). Pretreatment with the antioxidant NAC reversed BSO-induced cell death (Figure 5D) but not CPT-induced cell death (data not shown). Buthionine sulfoximine treatment increased autophagy levels, as indicated by increased LC3II levels, in autophagy-competent DLM8 cells (Figure 5E). N-acetyl cysteine treatment reversed CPT-induced and BSO-induced autophagy induction in autophagy-competent DLM8 cells (Figure 5F and G).

### Autophagy inhibition decreases CPT-induced mitochondrial membrane potential (ΔΨm) depolarization

**Figure 6:**
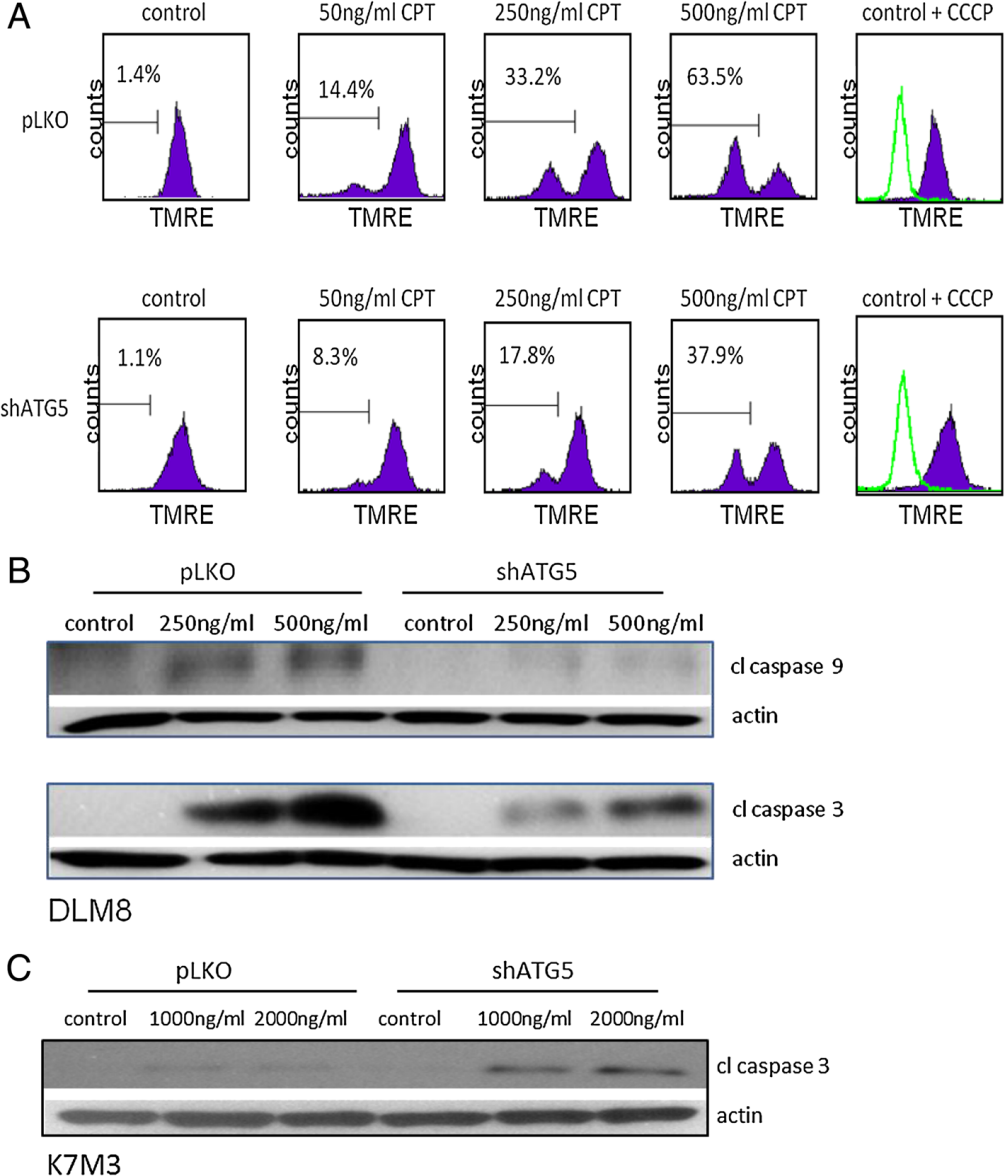
**CPT induces mitochondrial membrane potential and induces caspase-9 activation.** Tetramethylrhodamine, ethyl ester, perchlorate (TMRE) was used to determine mitochondrial membrane potential (∆Ψm). **A**, Mitochondrial membrane potential depolarization in autophagy-competent and autophagy-inhibited DLM8 cells following 24 h CPT treatment. Following 24 h CPT treatment, cells were incubated with TMRE followed by flow cytometry analysis. Decreased TMRE fluorescence is indicative of decreased ∆Ψm and increased release of pro-apoptotic molecules into the cytosol. Cells were incubated with the membrane uncoupler carbonylcyanide m-chlorophenylhydrazone (CCCP) prior to TMRE incubation to depolarize the mitochondrial membrane and serve as a positive control. Open histogram represents CCCP + TMRE treatment. Filled histogram represents cells incubated with TMRE only. Percentage values represent degree of mitochondrial membrane depolarization. Data is representative of results from two independent experiments. **B**, Caspase-9 activation and caspase-3 activation is reduced in autophagy-inhibited DLM8 cells. **C**, Caspase-3 activation is increased in autophagy-inhibited K7M3 cells. Cells were treated with CPT for 48 h. Cells were next collected, lysed and total protein immunoblotted for cleaved caspase-9 or cleaved caspase-3. Actin served as a protein loading control. Immunoblots are representative of immunoblots from at least two independent experiments.

Previously reported CPT-induced mitochondrial damage [14] prompted an investigation into the impact of autophagy inhibition on mitochondrial damage following CPT treatment. Camptothecin induced mitochondrial damage in both autophagy-competent and autophagy-inhibited DLM8 cells as determined by ΔΨm depolarization. However, ΔΨm depolarization was greater in autophagy-competent DLM8 cells compared to autophagy-inhibited DLM8 cells (Figure 6A), suggesting that mitochondrial damage was less in autophagy-inhibited DLM8 cells following CPT treatment. Caspase-9 activation and caspase-3 activation was greater in autophagy-competent DLM8 cells compared to autophagy-inhibited DLM8 cells following CPT treatment (Figure 6B). Caspase-3 activation was greater in autophagy-inhibited K7M3 cells compared to autophagy-competent K7M3 cells (Figure 6C).

## Discussion and conclusions

The protective role of autophagy induction against anticancer therapy is supported by observations that autophagy inhibition increases anticancer drug efficacy [2]. A literature search returned a limited number of studies reporting reduced anticancer therapy efficacy in autophagy-inhibited cells [15]. With autophagy inhibition currently being investigated as adjuvant anticancer therapy, these limited observations are relevant. In this study, ATG5 protein expression was knocked down to inhibit autophagy. Here, we report an opposing effect of ATG5 knockdown-mediated autophagy inhibition on CPT-induced cytotoxicity within OS. Autophagy inhibition decreased sensitivity to CPT in DLM8 cells and increased sensitivity to CPT in K7M3 cells. To date, there are no reports showing an opposing impact of autophagy inhibition on anticancer therapy within OS.

Following the observation that autophagy inhibition in K7M3 cells increased sensitivity to CPT, we reasoned that autophagy plays a greater role in the overall maintenance and metabolic homeostasis in K7M3 cells and suspected that the basal level of autophagy in K7M3 cells is greater than that of DLM8 cells. Immunoblot analysis of LC3II confirmed that basal level of autophagy is higer in K7M3 cells compared to DLM8 cells and non-transformed murine MC3T3 osteoblasts (Figure 4E). This finding supports the suggestion that K7M3 cells have an increased dependence on autophagy for ordinary metabolic activities. The dependence of K7M3 on autophagy is further supported by the observation that autophagy inhibition significantly decreased both K7M3 cell metabolic activity and cell growth (Figure 4B and C). It is plausible that increased basal level of autophagy in K7M3 cells is one of several genetic influences that contribute to the cancer phenotype and decreased autophagic capability increases sensitivity to stresses such as anticancer treatment. Increased dependence on autophagy has been reported for other cancers. For example, pancreatic cancer cells [16] and Ras oncogenic-driven cancer cells [17] have been shown to have increased dependence on autophagy. These two studies also reported increased basal levels of autophagy.

In this study, autophagy inhibition decreased sensitivity to CPT in DLM8 cells, which contrasts the more often reported observation that autophagy inhibition increases sensitivity to anticancer drug treatment. Therefore, we were particularly interested in the response of autophagy-inhibited DLM8 cells to CPT and explored further this cell line. While it was clear that autophagy inhibition in DLM8 cells decreased CPT-induced cell death compared to autophagy-competent DLM8 cells, the mechanism was unknown. Considering that the mechanism of action for CPT is DNA damage [18], we explored the impact of autophagy inhibition on CPT-induced DNA damage as a possible mechanism for decreased sensitivity to CPT in autophagy-inhibited DLM8 cells. DNA damage as determined by phosphorylation of p53 at Ser15 [19] was unchanged between autophagy-competent and autophagy-inhibited DLM8 cells (Figure 4F). We also assessed the impact of autophagy inhibition on DLM8 cell growth. Autophagy inhibition did not significantly impact cell growth of DLM8 cells (Figure 1C). This is relevant because the mechanism of action for CPT is DNA damage that occurs during cell division. Had autophagy inhibition significantly reduced DLM8 cell growth, this would support the suggestion that autophagy inhibition-mediated protection is due to reduced cell division. Together, this set of data suggests that the autophagy inhibition-mediated protection observed in this study was not due to reduced DNA damage or reduced cell division.

Previous reports of CPT-induced oxidative stress [20] led us to investigate the impact of autophagy inhibition on CPT-induced oxidative stress as a contributing factor to the observed autophagy inhibition-mediated protection. Oxidative stress, as determined by generation of ^.^O_2_^-^ and H_2_O_2_, was higher in autophagy-competent DLM8 cells compared to autophagy-inhibited DLM8 cells following CPT treatment, indicating that autophagy inhibition decreased CPT-induced oxidative stress. Autophagy inhibition also reduced basal oxidative stress level. To our knowledge, this is the first report of autophagy inhibition-mediated reduced basal oxidative stress as well as autophagy inhibition-mediated reduced anticancer drug-induced oxidative stress.

Increased levels of CPT-induced oxidative stress coupled with increased CPT-induced cell death in autophagy-competent DLM8 cells led us to determine if autophagy-competent DLM8 cells are more sensitive to oxidative stress. The use of BSO allowed for the investigation of the impact of oxidative stress alone on cell death and autophgay induction. Autophagy-competent DLM8 cells were more sensitive than autophagy-inhibited DLM8 cells to BSO-induced cell death. In agreement with Martinez-Outschonnra et al. [21], BSO also induced autophagy. BSO-induced cell death and BSO-induced autophagy induction were reversed by NAC pretreatment indicating a link between increased oxidative stress and both cell death and autophagy induction.

Basal levels of autophagy have been previously reported to differ among cancer cell lines [17] and here we report varying basal levels of autophagy in two metastatic murine OS cell lines. Considering these reports, it is plausible that the threshold level of autophagy induction that causes autopahgic cell death also varies for different cancers or even different cell lines within the same type of cancer. Camptothecin-induced DNA damage and CPT-induced oxidative stress together may have caused autophagy induction in autophagy-competent DLM8 cells that exceeded the threshold level necessary to cause autophagic cell death. Considering this, autophagy inhibition would reduce or delay CPT-induced autophagic cell death, making autophagy-inhibited DLM8 cells less sensitive to CPT-induced cell death. Therefore, one explanation for decreased sensitivity of autophagy-inhibited DLM8 cells compared to autophagy-competent DLM8 cells is reduced CPT-induced autopahgic cell death. Camptothecin-induced oxidative stress was lower in autophagy-inhibited DLM8 cells compared to autophagy-competent DLM8 cells. Therefore, an alternative explanation for decreased sensitivity to CPT in autophagy-inhibited DLM8 cells is reduced oxidative stress-induced cell death unrelated to autophagic cell death. At this point in our investigation, we are unable to present data supporting an explanation for lower oxidative stress in autophagy-inhibited DLM8 cells. However, the endogenous antioxidant catalase is a reported target of selective autophagy [22] and we suspect that autophagy inhibition may affect levels of endogenous antioxidants.

With observed CPT-induced oxidative stress in this study and reports of oxidative stress induced mitochondrial damage [23], we investigated the impact of CPT on mitochondria. In agreement with a previous study [24], CPT caused ΔΨm depolarization in both autophagy-competent and autophagy-inhibited DLM8 cells (Figure 6A). However, ΔΨm depolarization was greater in autophagy-competent DLM8 cells, suggesting increased mitochondrial damage. Mitochondrial membrane potential depolarization and mitochondrial damage is associated with caspase-9 activation and caspase-3 activation [25]. Immunoblot confirmed increased caspase-9 activation and caspase-3 activation in autophagy-competent DLM8 cells compared to autophagy-inhibited DLM8 cells (Figure 6B). Thus, the observed mitochondrial damage was likely an upstream event of caspase activation and likely contributed to increased cell death in autophagy-competent cells. Conversely, caspase-3 activation was higher in autophagy-inhibited K7M3 cells compared to autophagy-competent cells (Figure 6C). This observation suggests that autophagy inhibition increased the sensitivity of K7M3 to CPT-induced apoptosis.

In conclusion, we show that autophagy inhibition can have an opposing impact on the response of OS cells following CPT treatment. Our data suggest that the protective mechanism of autophagy inhibition involves both reduced oxidative stress and mitochondrial damage. The results of this study reminds us that autophagy inhibition can decrease the efficacy of anticancer drug therapy and underscores the need to better understand and predict the response of autophagy-modulated cancer cells to anticancer drug therapy.

## Abbreviations

OS: Osteosarcoma; NAC: N-acetyl cysteine; BSO: buthionine sulfoximine; CPT: Camptothecin; HE: Dihydroethidium; DCFH-DA: 2′,7′-dichlorofluorescein diacetate; ATG5: Autophagy-related protein-5; AVO: Acidic vesicular organelle; ∆Ψm: Mitochondrial membrane potential.

## Competing interests

The authors declare that they have no competing interest.

## Authors’ contributions

MGH conceived the study, carried out experiments, carried out data analysis and wrote the manuscript. NG assisted with project conception. JMS assisted with experiments and preparation of manuscript. ESK served as project supervisor. All authors read and approved the final manuscript.

## Pre-publication history

The pre-publication history for this paper can be accessed here:

http://www.biomedcentral.com/1471-2407/13/500/prepub

## Supplementary Material

Additional file 1: Figure S1 Camptothecin treatment decreases p62 protein expression. Reduced p62 protein expression is indicative of autophagy induction. Wildtype DLM8 and K7M3 cells were treated with CPT for 48 h. Cells were next collected, lysed and 30ug of total protein immunoblotted for p62. Actin served as a protein loading control. Immunoblots are representative of immunoblots from at least two independent experiments. Click here for file

Additional file 2: Figure S2 Bafilomycin A1 treatment increases LC3II protein expression. Wildtype DLM8 and K7M3 cells were treated with Bafilomycin A1 to determine the functional status of autophagy. Bafilomycin A1 inhibits autophagosome and lysosome fusion causing an increase in LC3II accumulation. Wildtype DLM8 and K7M3 cells were treated with Bafilomycin A1 for 48 h. Immunoblots are representative of immunoblots from at least two independent experiments. Click here for file
