# Supplementary figures and images for: Knockdown of autophagy-related protein 5, ATG5, decreases oxidative stress and has an opposing effect on camptothecin-induced cytotoxicity in osteosarcoma cells
Source: BMC Cancer. 2013 Oct 26;13:500. doi: 10.1186/1471-2407-13-500 (PMC3924338; doi:10.1186/1471-2407-13-500)

Additional file 1: Figure S1


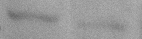

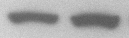


control 250ng/ml CPT

DLM8

p62

actin


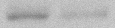

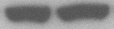


control 1000ng/ml CPT

p62

actin

K7M3

Supplement: Additional file 1: Figure S1 — Camptothecin treatment decreases p62 protein expression. Reduced p62 protein expression is indicative of autophagy induction. Wildtype DLM8 and K7M3 cells were treated with CPT for 48 h. Cells were next collected, lysed and 30ug of total protein immunoblotted for p62. Actin served as a protein loading control. Immunoblots are representative of immunoblots from at least two independent experiments. [file 1471-2407-13-500-S1.doc]
