# Supplementary material for: Knockdown of autophagy-related protein 5, ATG5, decreases oxidative stress and has an opposing effect on camptothecin-induced cytotoxicity in osteosarcoma cells
Source: BMC Cancer. 2013 Oct 26;13:500. doi: 10.1186/1471-2407-13-500 (PMC3924338; doi:10.1186/1471-2407-13-500)
Supplement: Additional file 2: Figure S2 — Bafilomycin A1 treatment increases LC3II protein expression. Wildtype DLM8 and K7M3 cells were treated with Bafilomycin A1 to determine the functional status of autophagy. Bafilomycin A1 inhibits autophagosome and lysosome fusion causing an increase in LC3II accumulation. Wildtype DLM8 and K7M3 cells were treated with Bafilomycin A1 for 48 h. Immunoblots are representative of immunoblots from at least two independent experiments. [file 1471-2407-13-500-S2.doc]

Additional file 2: Figure S2


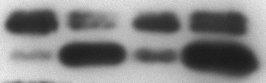

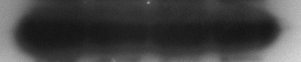


LC3I (16kDa)

LC3II (14kDa)

actin (42kDa)

control 5nM Baf1 50ng/ml CPT Baf1+CPT

DLM8


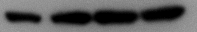

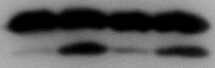


LC3I (16kDa)

LC3II (14kDa)

actin (42kDa)

control 5nM Baf1 50ng/ml CPT Baf1+CPT

K7M3
